# Rapid assessment of forest canopy and light regime using smartphone hemispherical photography

**DOI:** 10.1002/ece3.3567

**Published:** 2017-11-01

**Authors:** Simone Bianchi, Christine Cahalan, Sophie Hale, James Michael Gibbons

**Affiliations:** ^1^ School of Environment Natural Resources and Geography Bangor University Bangor Gwynedd UK; ^2^ Northern Research Station Forest Research Roslin Midlothian UK

**Keywords:** canopy openness, light regime, site factors, total gap fraction

## Abstract

Hemispherical photography (HP), implemented with cameras equipped with “fisheye” lenses, is a widely used method for describing forest canopies and light regimes. A promising technological advance is the availability of low‐cost fisheye lenses for smartphone cameras. However, smartphone camera sensors cannot record a full hemisphere. We investigate whether smartphone HP is a cheaper and faster but still adequate operational alternative to traditional cameras for describing forest canopies and light regimes.

We collected hemispherical pictures with both smartphone and traditional cameras in 223 forest sample points, across different overstory species and canopy densities. The smartphone image acquisition followed a faster and simpler protocol than that for the traditional camera. We automatically thresholded all images. We processed the traditional camera images for Canopy Openness (CO) and Site Factor estimation. For smartphone images, we took two pictures with different orientations per point and used two processing protocols: (i) we estimated and averaged total canopy gap from the two single pictures, and (ii) merging the two pictures together, we formed images closer to full hemispheres and estimated from them CO and Site Factors. We compared the same parameters obtained from different cameras and estimated generalized linear mixed models (GLMMs) between them.

Total canopy gap estimated from the first processing protocol for smartphone pictures was on average significantly higher than CO estimated from traditional camera images, although with a consistent bias. Canopy Openness and Site Factors estimated from merged smartphone pictures of the second processing protocol were on average significantly higher than those from traditional cameras images, although with relatively little absolute differences and scatter.

Smartphone HP is an acceptable alternative to HP using traditional cameras, providing similar results with a faster and cheaper methodology. Smartphone outputs can be directly used as they are for ecological studies, or converted with specific models for a better comparison to traditional cameras.

## 1 INTRODUCTION

Solar radiation is fundamental in forest ecosystems as it drives plant photosynthesis, morphogenesis, and fluxes of carbon, water, and energy between soil, vegetation, and the atmosphere (Ligot & Balandier, 2014). The analysis of the light intercepted by the tree crowns has been the basis for various ecological studies, especially for the dynamics of the vegetation growing under canopy cover (e.g., Coates, Canham, Beaudet, Sachs, & Messier, 2003; Duchesneau, Lesage, Messier, & Morin, 2001; Finzi & Canham, 2000; Pacala et al., 1996). Evans and Coombe (1956) started using hemispherical photography (HP) for light analysis in forest research after they discovered the “ingenious ‘fisheye’ camera” developed by Hill (1924) for cloud observations. Later, Anderson (1964a,b, 1966) made a crucial contribution to the computation of light transmittance through tree crowns using such photographs. HP is now considered the most widely used ground‐based method for describing both canopy characteristics and forest light regimes (Chianucci & Cutini, 2013; Promis et al., 2011). It is an indirect method for measuring the light transmittance with an associated level of error that can occasionally be substantial (Ligot & Balandier, 2014). However, its advantage over instantaneous light measurement is that its results do not inherently vary with time of day, time of year, or cloud cover. Direct measurements of light, such as quantum sensors, can be heavily affected by the conditions at the time of the observations (Anderson, 1966), require longer and more expensive data collection, and are more difficult to be linked to stand conditions (Čater, Schmid, & Kazda, 2013). Another photographic method used in forested environments is cover photography, which does not use a fisheye lens and is focused more on canopy parameters analysis such as the leaf area index (Chianucci & Cutini, 2013; Macfarlane, Grigg, & Evangelista, 2007).

Hemispherical photography is commonly implemented with analog or digital cameras equipped with 180° field‐of‐view (FOV) “fisheye” lenses pointing upward. The first processing step is to estimate the amount of sky visible through the canopy, by classifying each pixel of the photograph as belonging either to the sky or to any blocking element from the vegetation (canopy, leaf, branches, or stems) (Gonsamo, Walter, & Pellikka, 2011). This is usually carried out by thresholding the image, which is done by selecting a brightness value and considering the image pixels above this as belonging to the sky and below to vegetation. Thresholding can be manual, if the operator visually decides the best brightness value to use, or automatic, if software‐based techniques are applied to make the process objective and reproducible (Nobis & Hunziker, 2005). Photograph exposure, by affecting the quality of the image, can strongly affect the thresholding process (Rich, 1990). Specifically, overexposure can lead to overestimation of the sky fraction, but there are various methods available to tackle this issue (Beckschäfer, Seidel, Kleinn, & Xu, 2013).

From a thresholded HP image, various methodologies and software have been developed to estimate several variables, sometimes leading to a confusion in terminology (see Gonsamo, D'odorico, & Pellikka, 2013). For canopy structural characteristics, Canopy Openness (CO; usually defined as proportion of sky visible from a point) is one of the most common parameters estimated with this technology. The light transmittance of the canopy has been described largely using the Site Factor definition from Anderson (1966): the percentage of incident solar radiation at a given site compared to the total incident solar radiation in the open over the same period. This analysis requires the knowledge of the position of each gap on the hemisphere and the geographical location of the photograph so that the sun track can be superimposed onto the hemisphere.

Film handling and processing constraints slowed the widespread adoption of HP until digital photography and computer software become available, leading to an increase in the use of this methodology (Chianucci & Cutini, 2012). Today, another potential technological advance in this field is the availability of low‐cost fisheye lenses for smartphone and tablet cameras. One published case has already shown that for canopy cover analysis, the proportion of the forest floor covered by the vertical projection of the tree crowns (Korhonen, Korhonen, Rautiainen, & Stenberg, 2006), and smartphone HP is comparable to HP using traditional cameras (Tichý, 2015). However, that study involved the use of a specific smartphone app (GLAMA—Gap Light Analysis Mobile Application) that is useful for on‐the‐fly analysis in the field but less so for larger‐scale studies, due to reduced processing options. Another smartphone app, HabitApp (Deichmann, Hernandez‐Serna, Delgado C., Campos‐Cerqueira, & Aide, 2017; McDonald & McDonald, 2016), allows a quick analysis of canopy cover but again with limited processing options.

**Figure 1:**
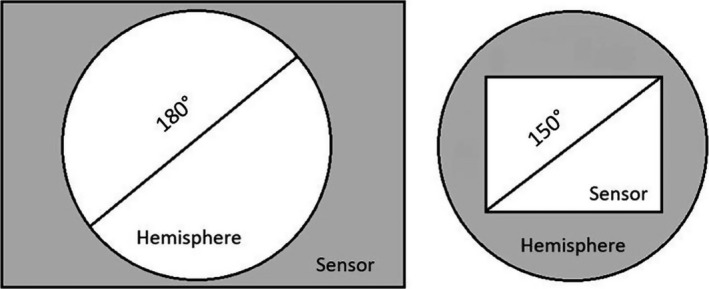
Circular hemispherical images with a full‐frame camera (left) versus diagonal smartphone hemispherical images (right). Adapted from Schneider et al. (2009)

Cameras traditionally employed for HP record circular photographs, while smartphone cameras take only diagonal photographs, following the definition of Schneider, Schwalbe, and Maas (2009) (Figure 1). Circular HP records the full hemisphere visible from the lens, while the diagonal photographs consider a smaller rectangular area. The fisheye lenses available for smartphones at the beginning of this study only provided a FOV of up to 160°, thus reducing even further the view compared to circular HP. Both these issues will surely lead to different estimations of CO between the cameras. The bias is expected to be toward higher values of openness in the smartphone HP as it excludes some of the peripheries of the image, the areas of the hemisphere usually more prone to be obscured. We are not aware of any studies where Site Factors are calculated from diagonal pictures. A sun track could be still laid on the pictures, but there will be portions of the hemisphere where the computation of the light transmittance will not be possible. However, in circular HP studies, the area at higher zenith angles (closer to the horizon) has sometimes been excluded from either CO or light transmittance computations, for exactly the reason that is more likely to be obscured (Machado & Reich, 1999) or because is prone to many sampling and optical errors (Gonsamo, Walter, & Pellikka, 2010). Sky areas located at the periphery have also less luminosity and a lower contribution to the Site Factor than areas located close to the zenith (Anderson, 1964a). Thus, it is possible that even if less accurate, smartphone diagonal HP could provide adequate information and in more quantity on both canopy structure and Site Factors, and, if a bias is present, it could be individuated and corrected. The challenge is to verify that the potential reduced accuracy of such measurements does not outweigh the benefits of using a cheaper, faster, less encumbering, more widespread technology with Internet connectivity. With smartphone HP, every forestry practitioner (or citizen scientists following the recent trends) could carry out quick canopy or light analysis without the need for extra tools other than a small fisheye lens that fits in a pocket. This could potentially lead to an amount of data substantially larger than in the traditional studies with smoothing of the probable errors present in the single measurements.

The main objective of the present research was to determine whether smartphone HP is an adequate operational alternative to traditional circular HP in describing canopy structural parameters and the light regime under canopy cover. For smartphone images, we will take two pictures with different orientations per sample point and use two processing protocols: (i) We estimate total canopy gap from the two single pictures, and average the values, and (ii) by merging the two pictures together, we form images closer to full hemispheres, so that we will be able to estimate from them CO and Site Factors as in circular HP. We verify whether smartphone values can be directly compared to circular HP ones, or, if a bias is present, whether models can be applied to transform and remove the bias. The values estimated from traditional circular HP images will be considered in our study the “ground‐truth” data against which we compare the smartphone HP estimates.

## 2 METHODOLOGY

### 2.1 Canopy and light parameter definitions

Of the various structural canopy parameters, we considered in this study: CO, the area fraction of the sky hemisphere that is unobstructed by canopy or other blocking elements when viewed from a single point; and Total Gap (TG), the ratio of the number of sky pixels to the total number of pixels in a hemispherical image (Gonsamo et al., 2011). The difference between the two parameters is that the CO calculation weights the gaps according to their position on the hemisphere, due to the geometric distortion produced by the fisheye lens (Gonsamo et al., 2011). This process assigns a lower weight to sky pixels located in the portions of the hemisphere with lower zenith angles, which are closer to the top of the hemisphere. For light regime measurements, we considered the Indirect Site Factor (ISF) as the transmittance through the canopy of the diffuse solar radiation generated by an overcast sky, the Direct Site Factor (DSF) as the transmittance of the direct solar radiation from a clear sky, and the Global Site Factor (GSF) as the total radiation that comprises both those components (Hale, Edwards, Mason, Price, & Peace, 2009). All the Site Factors were considered averaged over 1‐year period. ISF is thus independent of the location and orientation of the photograph: It is necessary only to know the zenith angle of the gaps (Anderson, 1966). To calculate DSF and subsequently GSF, a sun track is overlaid on the photograph to analyze how each gap interacts with the direct sunlight at different moments of the day and of the year (Anderson, 1964a). In all cases, the values range from zero (fully closed canopies and no light) to one (no canopy cover and full light).

### 2.2 Study sites

We collected data from 223 sample points distributed in 24 stands located in eight forests across the UK to consider different species, overstory, and geographical conditions (see Table 1). For each stand, we laid out ten sample points with a random systematic approach. We drew random transects on a desktop map and placed on them evenly spaced points, later identified in the field using a GPS receiver. The distance between points varied with the size of the stand. As most of the stands were originated by artificial planting, transects were not laid out parallel to each other to avoid following the planting lines. When carrying out the field survey, if a sample point fell in an open gap with no overstory, we relocated it under canopy cover if possible; otherwise, it was discarded (thus some stands had <10 sample points).

**Table 1:** Overview of the study sites

Forest	Location (WGS84)	Overstory type	Number of stands	Number of sample points
Clocaenog (Wales)	53°04′N, 3°24′W	Spruce	4	39
		Larch	1	10
Kielder (England)	55°13′N, 2°27′W	Spruce	4	37
Aberfoyle (Scotland)	56°13′N, 4°21′W	Larch	2	20
		Spruce	1	9
Treborth (Wales)	53°13′N, 4°10′W	Broadleaves	1	10
Newborough (Wales)	53°09′N, 4°20′W	Pine	2	20
Mortimer (England)	52°21′N, 2°45′W	Broadleaves	1	8
		Douglas	1	9
Coed‐Y‐Brenin (Wales)	52°48′N, 3°53′W	Douglas	2	17
Wykeham (England)	54°16′N, 0°33′W	Pine	4	36
		Spruce	1	8
Total			24	223

We assigned to each compartment a categorical variable named OV according to the overstory main species, with the following levels: “broadleaves” for mixed stands composed mainly of European beech (*Fagus sylvatica* L.) and oaks (*Quercus petraea* [Matt.] Liebl. and *Quercus robur* L.); “douglas” for Douglas fir (*Pseudotsuga menziesii* [Mirb.] Franco), sometimes associated with broadleaves; “larch” for European and Japanese larch (*Larix kaempferii* [Lamb] Carr. and *Larix decidua* Mill.); “pine” for Corsican and Scots pine (*Pinus nigra* subsp. *laricio* Maire and *Pinus sylvestris* L.); and “spruce” for Sitka spruce (*Picea sitchensis* [Bong.] Carr.).

### 2.3 Data collection

At each sample point, we took circular hemispherical color photographs in quick succession, under overcast sky or beneath a clear sky after sunset (Fournier, Landry, August, Fedosejevs, & Gauthier, 1996). We employed either a Nikon Coolpix 4500 or a Nikon Coolpix 990 equipped with Nikon FC‐E8 183° Fish‐Eye Converter Lens with azimuthal equidistant projection. Of the 223 sample points, in 145 we took hemispherical photographs at a fixed height of 130 cm, while in 78 points (the ones in Newborough, Mortimer, and Wykeham forests) we took them above a regenerating seedling or sapling which varied from 30 to 200 cm, as part of another research (data unpublished). The camera was positioned on a tripod and oriented to the north using a compass and upward to the zenith using a level. We took a picture using the automatic exposure and then three more with, respectively, −0.3, −0.7, and −1 exposure values (EV) to obtain at least one picture with good contrast between sky and canopy (Hale et al., 2009). The Nikon Coolpix 4500 recorded pictures of 2,048 × 1,536 pixels, the Nikon Coolpix 990 pictures of 2,272 × 1,704 pixels. Due to this difference, we had to keep the pictures separated during some of the processing steps, but the results (see later) did not differ between the two cameras, simply called “circular HP” from here onward.

In the same spot as each circular HP, and at the same height, we collected diagonal hemispherical color photographs with a Samsung Galaxy Grand Prime smartphone, equipped with a built‐in CMOS 8.0 MP camera and a 150° Aukey fisheye lens with azimuthal equidistant projection. We took the pictures immediately after reaching the point and with fewer precautions regarding the sky conditions (i.e., sometimes we waited for overcast sky conditions for the circular HP acquisitions, but never for the smartphone). We held the smartphone by hand, keeping it leveled and pointing upward as best as we could. We took two pictures, once aligning the smartphone north–south and once east–west with the aid of a compass, always using the automatic exposure. The smartphone pictures had pixel dimensions of 3,264 × 1,836. We purposely followed a faster protocol and used less equipment (no tripod and no level) for collecting the smartphone HP.

### 2.4 Image processing

We automatically classified all the circular HP images using two systems. The first was the Ridler and Calvard (1978) iterative selection method applied to the blue channel of the pictures, where differences between sky and vegetation pixels are most evident. We used this method with the function IsoData from the software Fiji (Schindelin et al., 2012). For the second method, we used the color‐based algorithm *enhanceHemiphoto* (from now on called EnhanceHP) from the package *Caiman* (Diaz & Lencinas, 2015) in R (R Core Team 2016). The EnhanceHP function combines the Ridler and Calvard (1978) method with a fuzzy pixel‐based classification based on the color attributes of hue, lightness, and chroma, working more efficiently where differences between sky and vegetation pixels are less evident. More documentation is available in Diaz and Lencinas (2015). We applied the CIMES‐FISHEYE software package (Gonsamo et al., 2011) to the outputs of both classification methods. We extracted the gap fraction information for each portion of the hemisphere with the function GFA, using a grid of 24 azimuth sectors and 18 zenith annuli. This information was the input for the following functions of the package: OPENNESS to obtain the CO, PARSOC for the ISFs (using the Standard Overcast Sky model), and PARCLR for the DSF. Using the same procedure as Hale et al. (2009), which in turn followed the recommendations of the Met Office (2006), we calculated the GSF as in Equation 1.

(1) GSF=0.65×ISF+0.35×DSF

We repeated the above estimations simulating a FOV of 150° by considering all the area comprised between the zenithal angles 75–90° as obstructed, and obtained the same parameters, named CO150, ISF150, DSF150, and GSF150.

For processing the smartphone pictures, we used two approaches. The first was to obtain TG separately from the east–west (E–W) and north–south (N–S) pictures in each sample point. After classifying each image with both the IsoData and EnhanceHP functions as above, we used the package *Raster* (Hijmans, 2016) of the R Statistical Software to calculate TG as the ratio of white pixels (gaps) to the total pixels. We estimated TG for both the N–S and E–W smartphone photographs, and then the average for each pair.

The second approach was to merge the two original pictures in each sample point and create a new one with the largest possible visible portion of the full hemisphere. We merged the images with the open source software “Hugin,” which automatically aligns and blends two or more images. The main use of Hugin is producing panoramic views, but we developed scripts to batch process our canopy photographs. Minor deviations from the N–S and E–W axes were frequent with the handheld smartphone, and we arbitrarily decided to use the E–W picture as the reference image for correct alignment. We thresholded all merged images with both the IsoData and Enhance function as above.

Using CIMES‐FISHEYE as above, we estimated COsm, ISFsm, DSFsm, and GSFsm (“sm” for smartphone) for each picture and each classification method. We carried out the calculations considering a full 180° FOV hemisphere, by setting up the GFA function of CIMES to extract the gap fraction of a larger circle than just the area covered by the merged images. Given that the diagonal length of one smartphone HP corresponds to 150°, we used a circle having a diameter equal to the diagonal length multiplied by the ratio 150°/180°. The software considered the portions of the hemisphere not covered by the merged images as obstructed (specifically, the area between the zenithal angles 75–90° and the corners not covered by merging the two pictures; in total around half of a full circular HP image. See online supplementary information for more details).

**Figure 2:**
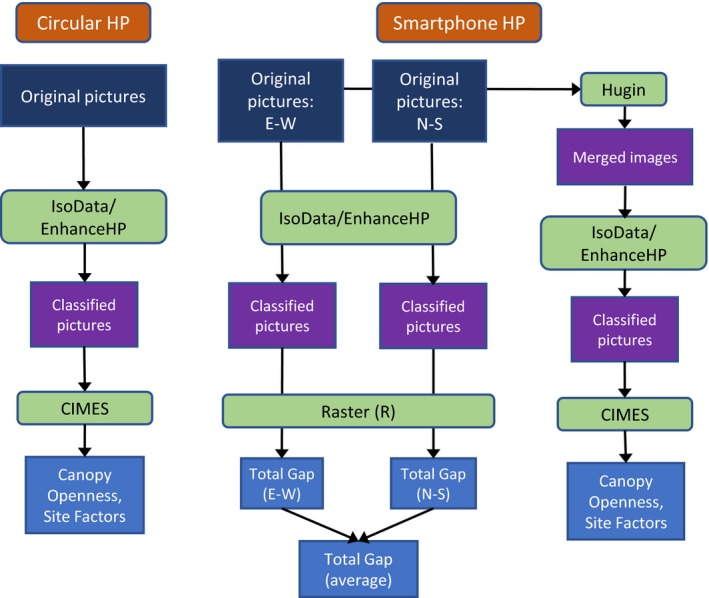
Simplified workflow of the various steps of image processing, from the original pictures to the output values

We carried out all the image processing with automatic and repeatable batch scripts. Figure 2 shows the workflow of the image processing. The original and merged pictures were in JPG format and were transformed during the thresholding into TIFF. The free software IrfanView was then used to batch convert all the files to BMP format for CIMES‐FISHEYE. The online Data S1 shows examples of the circular, single smartphone and merged smartphone HP images, highlighting the corresponding coverage.

### 2.5 Statistical analysis

To determine whether there were significant differences between the thresholding methods, we compared the TG and CO estimations of the two methods when applied to the same camera pictures. To assess the differences between the estimations from circular images when different FOVs were considered, we compared the respective CO and Site Factor estimations.

Then, we compared the following parameters estimated from the different cameras but using the same thresholding method: CO from circular HP images (only FOV 180°) and TG from smartphone HP images (both single orientation and average values); and CO, ISF, DSF, and GSF from circular HP images (only FOV 180°) and from merged smartphone HP images. We estimated generalized linear mixed models (GLMMs) of circular HP parameters as functions of the corresponding smartphone HP values. We tested as fixed effects the overstory type both as a main term and as an interaction term, to account for differences between species. We also included terms related to the different circular camera (“camera_type,” with the values of either “N990” or “N4550”) and the data collection methodology (“height_from_ground,” with the values of either “130 cm” or “variable”), to verify whether such differences were significantly affecting the relationship. We used a random effect of compartments nested within forests, to account for the sampling structure. From a global model including all the above effects, we then assessed reduced models with fewer effects using the Akaike information criteria (AIC), and selected the one with the lowest AIC as the best model for each analysis (Symonds & Moussalli, 2011). We carried out all analyses using the packages *nlme* (Pinheiro, Bates, DebRoy, & Sarkar, 2016) and *stats* in R (R Core Team 2016).

## 3 RESULTS

**Figure 3:**
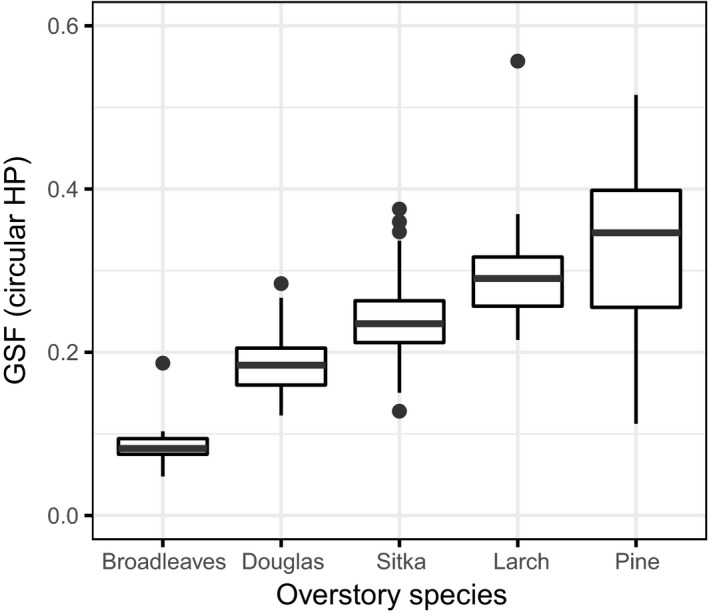
Boxplots of Global Site Factor (GSF) from circular hemispherical images for different overstory species. The horizontal line shows the median value, the boxes represent the values between the first and third quartiles, and the vertical lines are an additional 1.5 interquartile range above and below them

Figure 3 shows the value distribution for GSF calculated from the circular HP images, using the EnhanceHP method, to provide a reference for the range of data. The areas surveyed in this research varied from low light transmittance (GSF around 0.05) to medium–high level of transmittance (GSF around 0.60), with most of them falling in the range GSF 0.20–0.30. However, the range was not even across different overstory types.

### 3.1 Comparison of thresholding methods

Canopy parameters estimated from the pictures taken by the same camera (respectively, the averaged TG for smartphone and CO for circular HP images), but classified with the different methods, were slightly lower for the EnhanceHP method than for the IsoData (mean of differences, respectively, −0.023 for TG and −0.027 for CO, *p*‐value <.001 for both). This means that more pixels were classified as canopy elements with EnhanceHP. A visual analysis of the thresholded images confirmed that EnhanceHP correctly identified as vegetation many elements that were mistaken for sky by the IsoData method. That was true not only in the few obvious cases of high exposure images but also for small vegetation elements under good contrast. As all the following analyses showed better correlations between the values from the circular and smartphone cameras when EnhanceHP was applied to both rather than the IsoData method, we present here only the former. Additional results for the IsoData method can be found in the online Data S1.

### 3.2 Comparison of different FOVs for circular HP

Values of CO, DSF, and GSF when estimated from circular HP images with FOV 150° were significantly lower than from FOV 180° (*p* < .001) although the difference was very small in absolute terms: the mean of the differences between the different FOV estimations was, respectively, −0.001 (*SD*, 0.009), −0.013 (*SD*, 0.022), and −0.004 (*SD*, 0.010). No significant difference was present for ISF.

### 3.3 Comparison of circular HP with nonmerged smartphone HP

**Figure 4:**
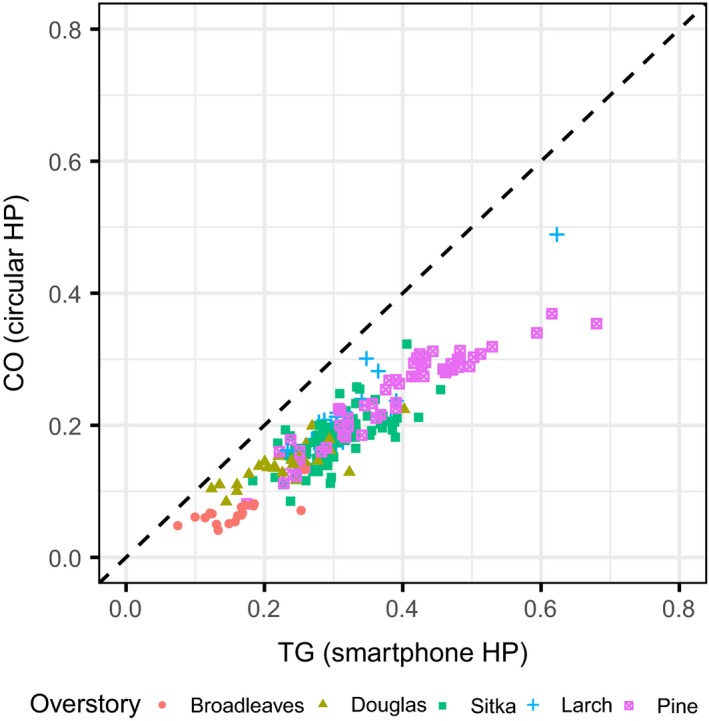
Scatterplot of Canopy Openness (CO) from circular image with Total Gap (TG) from smartphone, showing the line of identity (dashed black line), both estimated using the EnhanceHP method. Smartphone values were obtained by averaging the single images results for each plot

The comparison of CO from circular HP images and TG from smartphone HP images (averaged between the two pictures), using the EnhanceHP method, is shown in Figure 4. TG values from the smartphone pictures were higher than CO values from circular HP images: mean of differences 0.12, *SD* 0.04. In relative terms, TG values from the smartphone pictures on average were 165% of the CO values from circular HP images. The GLMM structure with lowest AIC maintained overstory type only as interaction term, while both the differences in the circular camera type and the height from the ground did not affect the relationship. See Table 2 for the AIC comparison between model structures and Table 3 for more details of the selected model. The effect of the overstory type was that for the same increase in the values of observed TG, the predicted CO values increased more rapidly for larch and pine than for broadleaves, with Sitka spruce and Douglas fir having an intermediate effect.

**Table 2:** Akaike information criteria comparison between different generalized linear mixed model structures for all analyses

Model	CO ~ TG	CO ~ Cosm	ISF ~ ISFsm	DSF ~ DSFsm	GSF ~ GSFsm
*y* ~ *x *+ *x*:OV* *+ OV* *+ camera* *+ HFG	−984	−980	−852	−761	−877
*y* ~ *x *+ *x*:OV* *+ OV* *+ camera	−991	−988	−861	−768	−885
*y* ~ *x *+ *x*:OV* *+ OV	−999	−997	−869	−776	−894
*y* ~ *x *+ *x*:OV	−**1,019**	−**1,016**	−**891**	−792	−**916**
*y* ~ *x *+ OV	−990	−980	−869	−780	−894
*y* ~ *x*	−1,009	−997	−885	−**795**	−908

TG is Total Gap, CO is Canopy Openness, and ISF, DSF, and GSF are, respectively, Indirect Site Factor, Direct Site Factor, and Global Site Factor (“sm” for smartphone HP). In the formulas, *y* and *x* are the respective circular HP and smartphone HP parameter considered, OV is the overstory type, camera is the type of Nikon Coolpix used for circular images, and HFG is the height from the ground at which the pictures were taken (see Methodology). The lowest AIC values are shown in bold.

**Table 3:** Results of generalized linear mixed models between the outputs estimated by circular and smartphone HP pictures, using the EnhanceHP method

Fixed effects	CO ~ TG			CO ~ COsm			ISF ~ ISFsm			DSF ~ DSFsm			GSF ~ GSFsm		
	Value	*SE*	*p*‐value	Value	*SE*	*p*‐value	Value	*SE*	*p*‐value	Value	*SE*	*p*‐value	Value	*SE*	*p*‐value
(Intercept)	0.025	0.010	.013	0.032	0.009	.001	0.042	0.012	.000	0.049	0.011	.000	0.049	0.011	.000
*x*	0.275	0.082	.001	0.309	0.118	.009	0.355	0.095	.000	0.764	0.046	.000	0.292	0.104	.002
*x*:OV(douglas)	0.197	0.080	.014	0.386	0.120	.002	0.347	0.094	.000	–	–	–	0.376	0.107	.001
*x*:OV(sitka)	0.228	0.081	.005	0.437	0.112	.000	0.341	0.086	.000	–	–	–	0.384	0.097	.000
*x*:OV(larch)	0.398	0.083	.000	0.694	0.118	.000	0.471	0.090	.000	–	–	–	0.542	0.101	.000
*x*:OV(pine)	0.267	0.081	.001	0.610	0.113	.000	0.487	0.088	.000	–	–	–	0.560	0.096	.000

Random effects (intercept)	*SD*			*SD*			*SD*			*SD*			*SD*		
Forest	0.018			0.012			0.008			0.000			0.009		
Stand (in Forest)	0.011			0.012			0.019			0.038			0.019		
Residual	0.020			0.020			0.031			0.034			0.028		

CO is Canopy Openness, TG is Total Gap, and ISF, DSF, and GSF are, respectively, Indirect Site Factor, Direct Site Factor, and Global Site Factor (“sm” for smartphone outputs). For the fixed effects, “*x*” indicates the smartphone HP parameter used in the model, and OV is the overstory type.

The TG values from smartphone pictures taken with different orientation in the same point, both classified with EnhanceHP, were not statistically significant (*p* = .53). However, when we used the TG values estimated only from the E–W and N–S pictures, instead of the averages, in the above model the results were slightly less accurate in both cases, although better for the E–W than the N–W pictures (results not shown).

### 3.4 Comparison of merged Smartphone HP with circular HP

**Figure 5:**
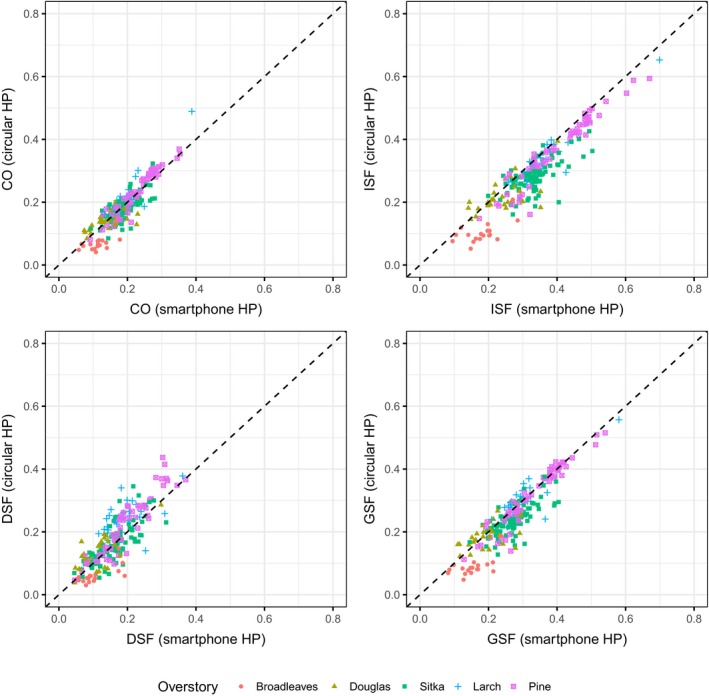
Scatterplots of Canopy Openness and Site Factors (respectively, ISF for Indirect, DSF for Direct, and GSF for Global Site Factor) estimated from different cameras, using EnhanceHP method, showing the line of identity (dashed black line). Smartphone values were obtained from merged images

The comparisons between the outputs estimated from the circular and the merged smartphone HP images, using the EnhanceHP method, are shown in Figure 5. The smartphone values were on average significantly different from the circular ones (*p* < .05 in all cases): mean of differences, respectively, 0.004 for CO (*SD*: 0.031), 0.042 for ISF (*SD*: 0.037), −0.012 for DSF (*SD*: 0.047), and 0.023 for GSF (*SD*: 0.040). In relative terms, the smartphone values on average were, respectively, the 102% (for CO), 115% (for ISF), 93% (for DSF), and 109% (for GSF) of the values of the circular HP values. For the CO, ISF, and GSF models, the GLMM structure with lowest AIC maintained overstory type as interaction term, while for the DSF both the main term and interaction term were dropped. In all cases, the differences in the circular camera type and the height from the ground did not affect the relationship. See Table 2 for the AIC comparison between model structures and Table 3 for more details of the selected models. When the effect of the overstory type was present, it meant again that for same increase in the values of observed smartphone HP values, the predicted circular HP values increased more rapidly for larch and pine than for broadleaves, with Sitka spruce and Douglas fir having an intermediate effect.

## 4 DISCUSSION

The results of the study suggest that smartphone‐based HP can be used as a faster and cheaper alternative to traditional camera sets. We demonstrate methods to obtain canopy structural parameters and Site Factors with the advantage of less expensive equipment and faster data collection time. We purposely carried out the smartphone image acquisition with a simpler protocol that does not need extra tools (such as a tripod or a level) or to wait for the best sky conditions. The rationale was to test a methodology that could be applied by any forest practitioner in a speedier way, potentially obtaining a higher amount of data. In this case study, a smartphone is used only for the image acquisition, while the processing is carried out subsequently in a computer. Thus, for example, in a crowd‐sourcing project, various operators can acquire the images in the field and, using other smartphone applications, upload them to a central server where the more advanced processing here described can take place.

While we carried out the smartphone pictures acquisition with fewer precautions, generally the images showed an acceptable quality in terms of exposure and contrast between sky and canopy and in turn the thresholding process gave good results. This is likely due to a combination of factors. The new sensors and in‐camera processing of the smartphones are likely better than the now almost 20‐year‐old Nikon Coolpix. The smaller FOV of the smartphone fisheye lens may have reduced the direct sunlight hitting the sensor. The generally favorable sky conditions of the UK (high latitude, cloudy climates) have likely also played an important role, so that in other geographical areas, the same precautions regarding direct sunlight may have to be applied also to smartphone HP. However, where suboptimal contrast between sky and canopy occurred in some of our smartphone pictures, the EnhanceHP function from the *Caiman* package gave good results during the thresholding. This method was designed to work with suboptimal images, while the IsoData function requires good contrast pictures.

The small differences between parameters estimated from circular HP images with a FOV of 150° and 180° demonstrate that the reduced FOV of the smartphone fisheye lens could not be the main source of difference between the two cameras, which most likely are the diagonal character of the camera sensors and the lower quality of the images. New smartphone camera sensors and lenses are likely to be developed continuously, influencing both issues due to changes in the resolution of sensors and the quality and FOV of the lens, and then in turn affecting the analyses carried out in this study with our particular combination of smartphone and fisheye lens. However, given that the same fisheye lens is used, the smartphone camera used can be considered representative of the average sensor resolution and quality nowadays available, and if only new sensors will have likely better characteristics. In any case, we suggest verifying the real FOV of the conversion lens.

Total Gap, obtained from the simple processing protocol of single smartphone pictures, was consistently higher than the CO values from circular hemispherical images, as expected. The bias between those values in this study was consistent and with a reduced deviation, suggesting that there is still potential to use TG from smartphone pictures in ecological study as a substitute for traditional circular camera analysis, either as it is or transformed using the model provided. Taking two pictures in the same point and averaging the results improved the results without significantly increasing the time required for data collection and processing, so we advise this operation for future studies.

Through the more advanced merging protocol, we obtained processed smartphone pictures that could be used for estimation of CO and Site Factors. The mean differences and *SDs* between the parameters from different cameras were relatively small. This suggests that the smartphone camera outputs could be used in place of those from a circular camera. As already discussed, the areas close to the horizon not covered by the smartphone HP images did not greatly affect the CO and ISF estimation. However, the different coverage was expected to give poorer results in the estimation of DSF, which is a function also of the location of the gaps in relation to the sun track. Particular gaps with a large contribution to this Site Factor in circular hemispherical images might be excluded from merged smartphone images. In addition, the handheld alignment of the smartphone in the field is likely to have introduced additional errors in the sun track overlay. However, for the DSF, the mean difference between the circular and smartphone cameras was even lower than for other parameters. For the GSF, which in the UK depends more from the Indirect than DSF, the differences between cameras were similar to the former. The best model structures for CO, ISF, and GSF included the overstory type as interaction term, that is, the relationship was affected by the different species’ foliar and crown architecture. Overstory type was not included in the model for DSF, which is likely more affected by large gaps falling around the sun track, and less by the overall fine gap structure. However, there were few replicates for some classes (i.e., only two broadleaved stands out of 24), and the range of CO sampled within classes was not equal (i.e., for broadleaved stands, it was lower than for pine and larch stands).

In conclusion, we believe that the cheaper and faster methodologies here described for smartphone‐based HP provide reliable parameters that can be used as substitutes for those estimated from circular cameras. Smartphone outputs could be employed as they are in forest ecology studies, such as for assessment of different sites or as inputs for ecological modeling, or converted with specific transformation models for a better comparison between cameras. The range of application of the models provided here outside the forest and sky conditions and smartphone specifications considered in this study has not been tested. As we first designed this study, new smartphone fisheye lenses promising wider angles (up to 180° and even more) are available on online marketplaces, providing different but more accurate results when applying the methodologies here described. Due to rapid technological development, smartphone HP could potentially gain increasing importance in future years.

## CONFLICT OF INTEREST

None declared.

## DATA ACCESSIBILITY

The dataset used for this study is stored on the Dryad Digital Repository https://doi.org/10.5061/dryad.f6506 together with the main scripts used for the processing of the smartphone hemispherical images with R Statistical software and Hugin.

## AUTHORS' CONTRIBUTION

Simone Bianchi designed the original work and methodology, carried out the data acquisition, analysis and interpretation, and prepared the manuscript. Christine Cahalan and Sophie Hale critically contributed to the data interpretation and manuscript revision. James Gibbons critically contributed to the methodology development, the data analysis and interpretation, and manuscript revision.
