# Supplementary material for: Rapid assessment of forest canopy and light regime using smartphone hemispherical photography
Source: Ecol Evol. 2017 Nov 1;7(24):10556–66. doi: 10.1002/ece3.3567 (PMC5743530; doi:10.1002/ece3.3567)
Supplement: Supplementary file 1 [file ECE3-7-10556-s001.docx]

Supplementary figures


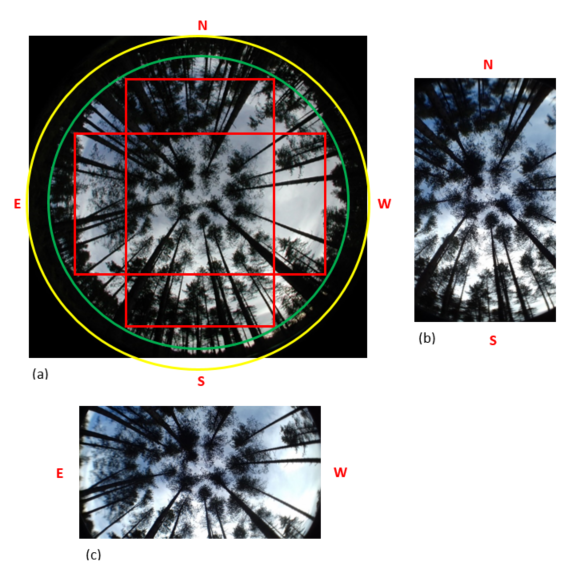


Figure S 1. Examples of the circular picture (a) and of the two smartphone pictures (b and c) for the same plot. The yellow circle represents the 183° FOV of the Nikon Coolpix. The green circle is the 150° FOV of the smartphone. The red rectangles show the approximate areas on the Nikon picture covered by each Smartphone picture. Note that in hemispherical pictures the East and West directions are reversed.


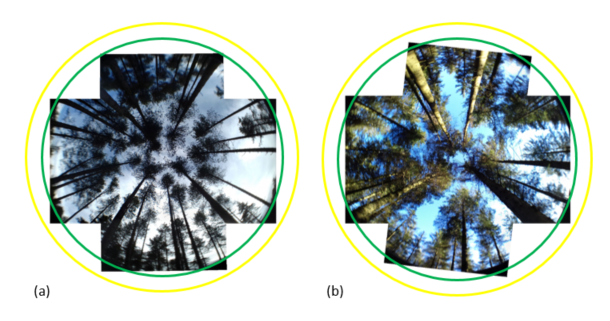


Figure S 2. Examples of the results of the merging process with Hugin for two plots. The green circles represent the approximate 150° FOV, while the yellow the 180° FOV. The left images were taken with a correct 90° angle from each other in the field, the right with a slight deviation. White pixels outside the merged images and within the yellow circles correspond to the area considered as blocking elements for the parameters estimations.


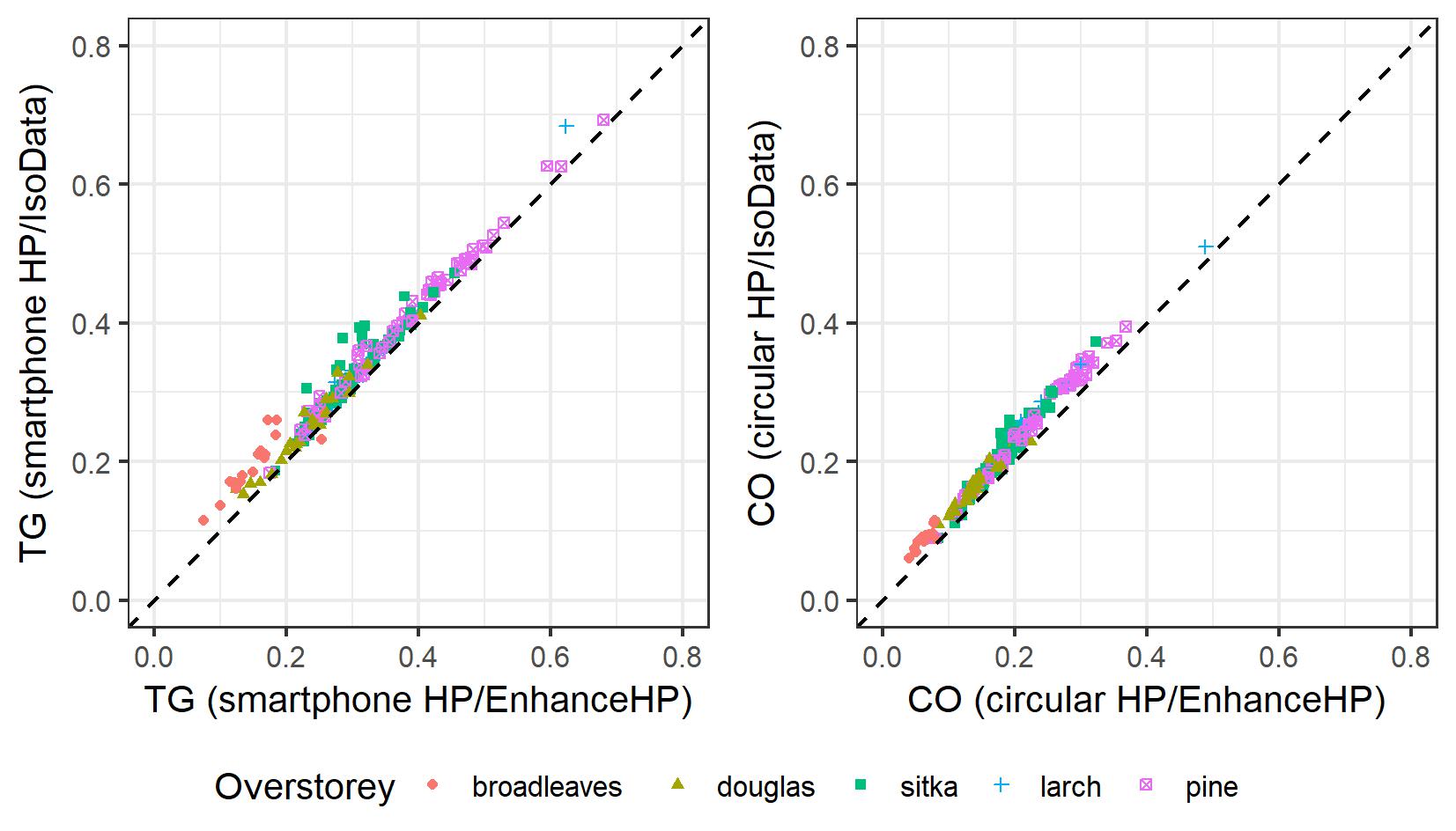


Figure S 3. Scatterplots of Total Gap (TG, left) and Canopy Openness (CO, right) from same camera pictures but with different classification methods, showing the line of identity (dashed black line).


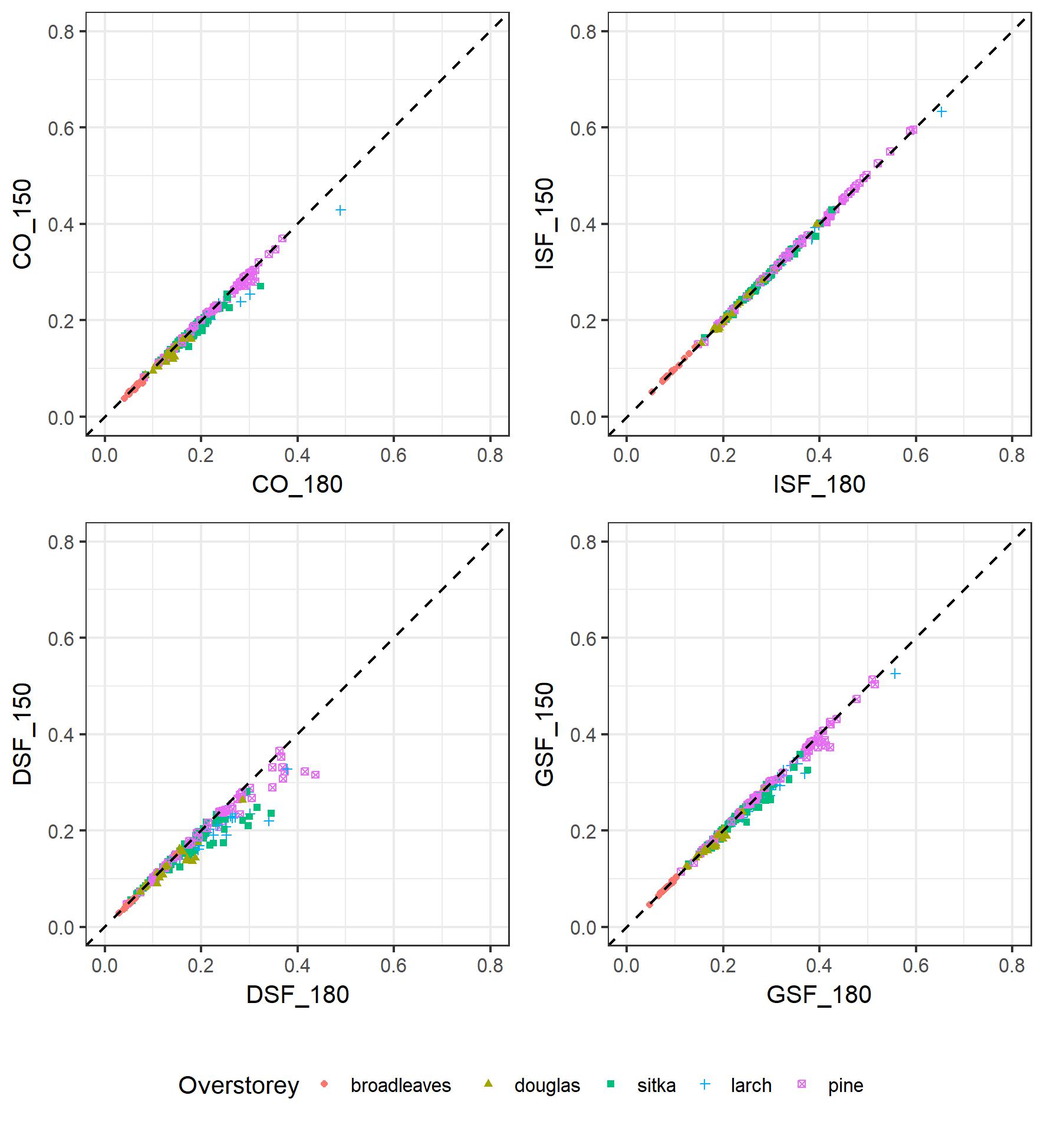


Figure S 3. Scatterplots of Canopy Openness and Site Factors (ISF, Indirect; DSF, Direct; GSF, Global) estimated from circular HP images with a Field of View of 150° (y-axis) and 180° (x-axis), showing the line of identity (dashed black line).


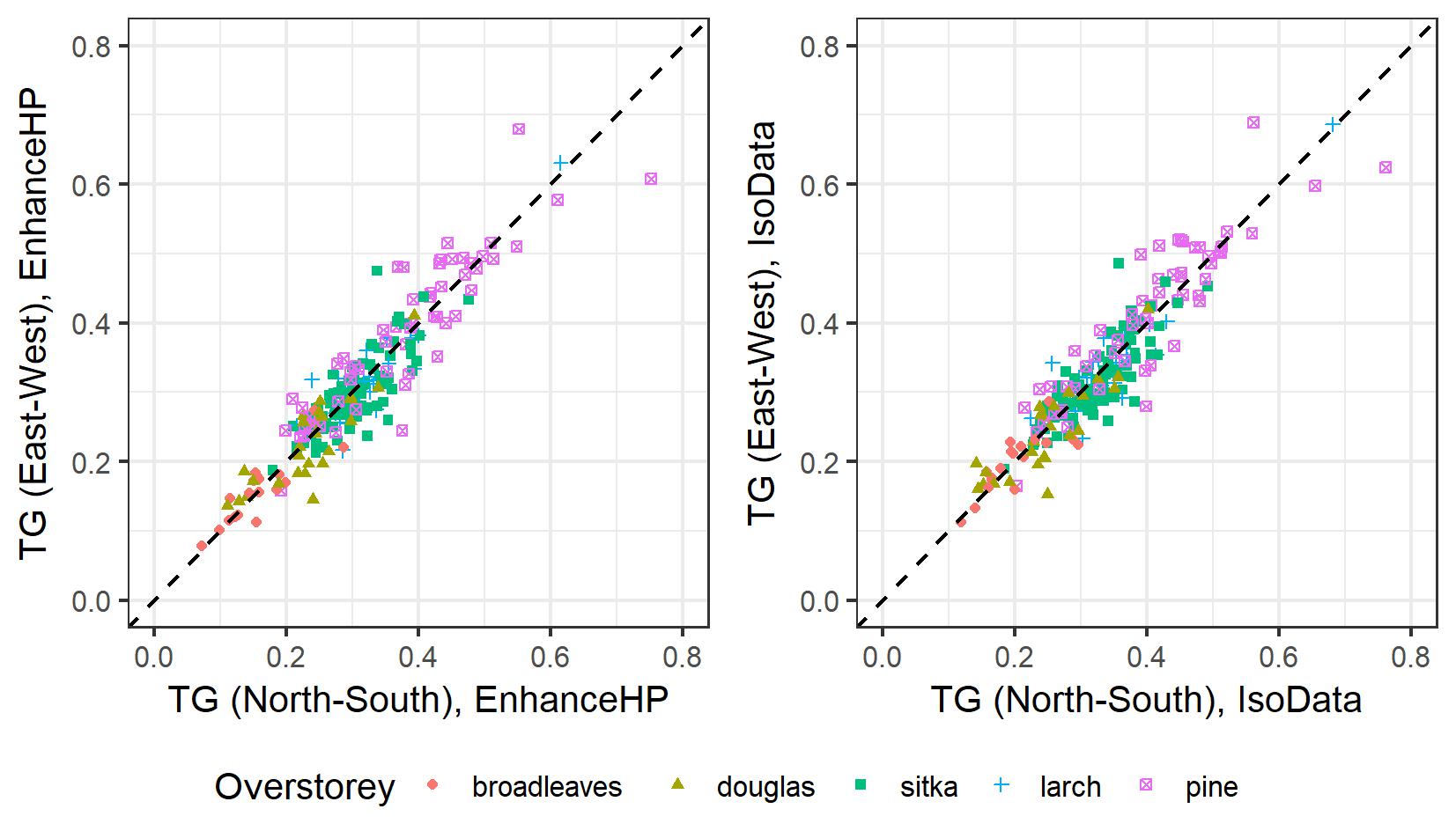


Figure S 4. Scatterplots of Total Gap (TG) from East-West (E/W) and North-South (N/S) oriented Smartphone pictures with the same classification method (left, EnhanceHP; right, IsoData), showing the line of identity (dashed black line).


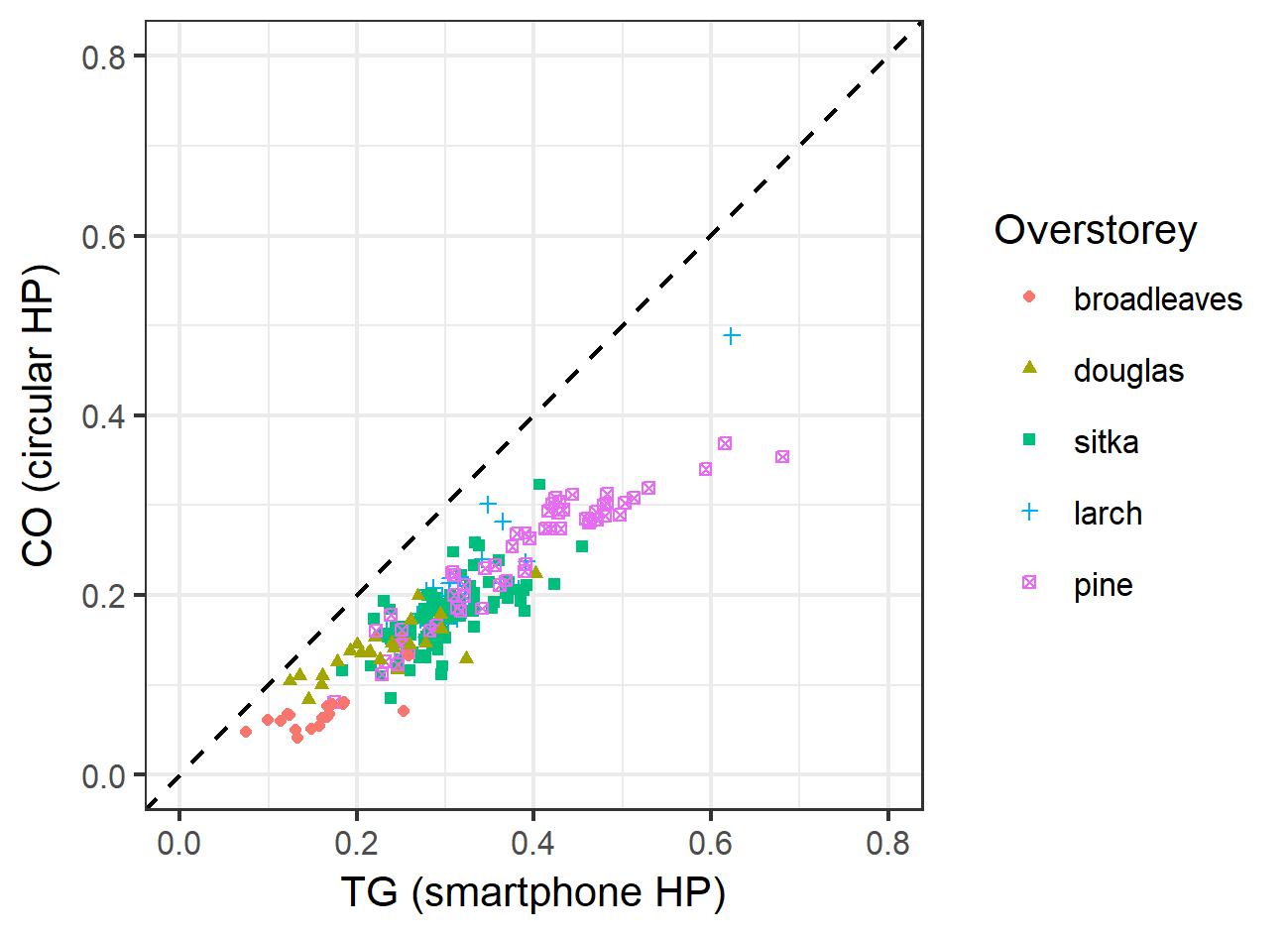


Figure S 5. Scatterplot of Canopy Openness (CO) from circular images and Total Gap (TG) from smartphone images, using IsoData method, showing the line of identity (dashed black line).


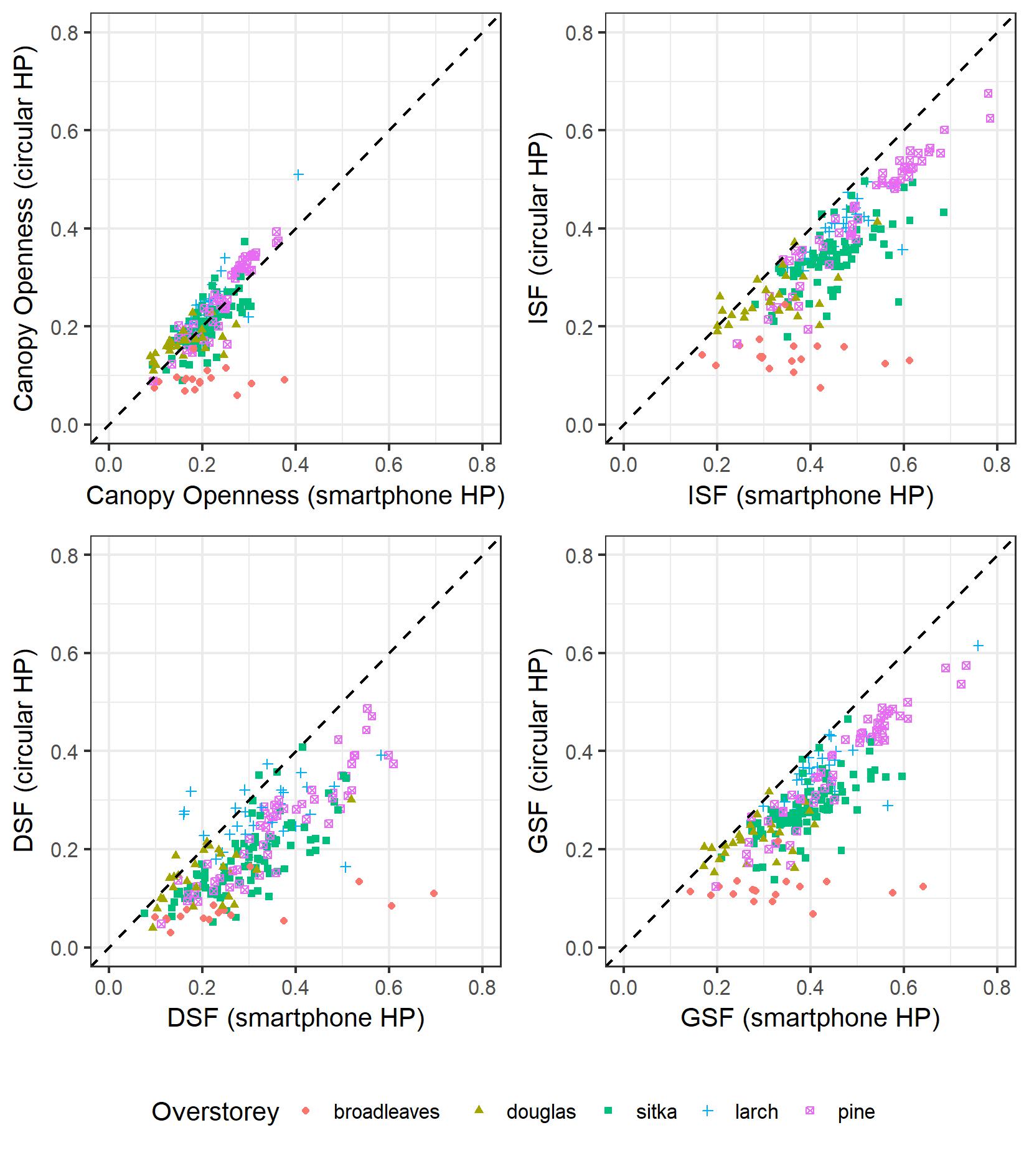


Figure S 6. Scatterplots of Canopy Openness and Site Factors (ISF, Indirect; DSF, Direct; GSF, Global) from Smartphone merged images versus the same values from the circular Images, using IsoData method. The dashed black line is the line of identity.
