# Supplementary material for: Rapid assessment of forest canopy and light regime using smartphone hemispherical photography
Source: Ecol Evol. 2017 Nov 1;7(24):10556–66. doi: 10.1002/ece3.3567 (PMC5743530; doi:10.1002/ece3.3567)
Supplement: Supplementary file 2 [file ECE3-7-10556-s002.docx]

Data S1. Supplementary information

Examples of smartphone hemispherical pictures

Figure S1 shows a circular hemispherical picture and the two smartphone hemispherical pictures in the same plot, providing guides to compare the respective coverages. Note that the Nikon Coolpix hemispherical photography famously excludes a small portion on the bottom of the pictures, while the smartphone hemispherical photography employed here sometimes had a diagonal field-of-view slightly smaller than the frame of the picture (see the black corners). In both cases, those areas were considered by the software as blocking elements.

Figure S2 shows the results of the merging process for the smartphone images in two plots. In the left picture, we took the two smartphone pictures with the correct alignment in the field, with angle of 90° from each other. In the right picture, we took the images with a slightly wrong alignment but the Hugin software automatically corrected the error. Note that the area with missing information within the yellow circle was considered as obstructed view.

Additional statistical results

Comparison of classification methods

The comparisons between outputs estimated from the same camera (respectively Total Gap from smartphone and Canopy Openness from circular images) but with different thresholding methods are shown in Figure S3.

Comparison of different FOVs for circular HP

The comparisons of parameters estimated from circular HP images with FOV 150° and 180° are shown in Figure S4.

Comparison of smartphone picture with different orientation

The comparisons between Total Gap estimated from smartphone pictures taken with different orientation, using the same classification methods, are shown in Figure S5.

Comparison of non-merged smartphone HP with circular HP, IsoData method

The comparison of Canopy Openness from circular pictures and Total Gap from smartphone values (averaged between the two pictures), using the IsoData method, is shown in Figure S6. CO from circular pictures was significantly higher (p < 0.001) than TG values from the smartphone pictures (mean of differences 0.115, st.dev. 0.04).

Comparison of stitched Smartphone HP with circular HP, IsoData method

The comparisons between the outputs estimated from the circular and the merged smartphone images, using the IsoData method, are shown in Figure S6. There were some plots where the IsoData thresholding applied to the merged smartphone pictures seems to have completely failed, resulting in differences between circular and smartphone values of around 0.3 and more. Even not considering these exceptions, the distribution of circular and smartphone values was generally worse for all parameters than when using EnhanceHP.
